# A Comprehensive Pan-Cancer Analysis for Pituitary Tumor-Transforming Gene 1

**DOI:** 10.3389/fgene.2022.843579

**Published:** 2022-02-25

**Authors:** Siming Gong, Changwu Wu, Yingjuan Duan, Juyu Tang, Panfeng Wu

**Affiliations:** ^1^ Department of Orthopaedics, National Clinical Research Center of Geriatric Disorders, Xiangya Hospital of Central South University, Changsha, China; ^2^ Institute of Anatomy, University of Leipzig, Leipzig, Germany; ^3^ Faculty of Chemistry and Mineralogy, University of Leipzig, Leipzig, Germany

**Keywords:** PTTG1, prognosis, immune infiltration, enrichment analysis, big data, pan-cancer analysis

## Abstract

Pituitary tumor-transforming gene 1 (PTTG1) encodes a multifunctional protein that is involved in many cellular processes. However, the potential role of PTTG1 in tumor formation and its prognostic function in human pan-cancer is still unknown. The analysis of gene alteration, PTTG1 expression, prognostic function, and PTTG1-related immune analysis in 33 types of tumors was performed based on various databases such as The Cancer Genome Atlas database, the Genotype-Tissue Expression database, and the Human Protein Atlas database. Additionally, PTTG1-related gene enrichment analysis was performed to investigate the potential relationship and possible molecular mechanisms between PTTG1 and tumors. Overexpression of PTTG1 may lead to tumor formation and poor prognosis in various tumors. Consequently, PTTG1 acts as a potential oncogene in most tumors. Additionally, PTTG1 is related to immune infiltration, immune checkpoints, tumor mutational burden, and microsatellite instability. Thus, PTTG1 could be potential biomarker for both prognosis and outcomes of tumor treatment and it could also be a promising target in tumor therapy.

## Introduction

Pituitary tumor-transforming gene 1 (PTTG1) encodes a protein which is involved in many cellular processes (Caporali et al., 2017; Lin et al., 2019a; Zhi et al., 2019). PTTG1, also known as human securin, plays a major role in the regulation of mitosis, especially during sister chromatid separation (Hatcher et al., 2014). Additionally, the PTTG1 protein engages in various other cellular processes, such as DNA damage/repair, apoptosis, and metabolism (Vlotides et al., 2007). According to previous studies, PTTG1 is normally viewed as an oncogene, and high expression of PTTG1 can promote tumorigenesis in many kinds of tumors such as prostate and bladder tumors (Wondergem et al., 2012; Xiang et al., 2017; Cui et al., 2020; Fraune et al., 2020). In particular, PTTG1 is one of the 17 gene signatures that can predict metastasis and prognosis in various tumor types (Ramaswamy et al., 2003). Some previous experiment-based studies have also indicated that PTTG1 is involved in various tumor processes, such as growth and metastasis, by revealing that PTTG1 overexpression is linked to proliferation and invasiveness (Zhang et al., 2014; Yan et al., 2015; Lin et al., 2016; Meng et al., 2020). Interestingly, the downregulation or silencing of PTTG1 could lead to the opposite result (Caporali et al., 2012; Cui et al., 2020; Meng et al., 2020; Zhou et al., 2020).

It has been shown that PTTG1 is involved in various molecular mechanisms such as epithelial-mesenchymal transition (EMT), PI3K/AKT signaling, and mitogen-activated protein kinase (MAPK) signaling pathways (Lin et al., 2016; Feng et al., 2017; Huang et al., 2018; Hu et al., 2019; Cui et al., 2020). EMT is the process by which epithelial cells may lose their own features, such as polarity and adherence ability, and their cellular migration and invasion ability are enhanced. EMT is a natural procedure in wound repair and inflammation; however, EMT could also be a critical process that could lead to tumor formation (Suarez-Carmona et al., 2017). MAPK, which is a member of the serine-threonine kinase family, is linked to cell proliferation, while PTTG1 is the regulator of mitosis (Fang and Richardson, 2005; Hatcher et al., 2014). Growing evidence has revealed that the MAPK pathway is associated with many processes, such as tumor formation, invasion, and other tumor-related behaving (Burotto et al., 2014; Sun et al., 2019; Lee et al., 2020). In addition, PTTG1 could also be a potential target in tumor therapy for various types of cancer, such as melanoma, breast cancer, and ovarian cancer (Nakachi et al., 2016; Caporali et al., 2017; Meng et al., 2020). PTTG1 is also involved in mitosis and linked to tumor formation, prognosis, and cancer treatment in various tumors. Our previous study showed that the regulator of chromatin condensation 1 (RCC1), which is also involved in the cell cycle, plays a key role in human pan-cancer (Wu et al., 2021). The PTTG1 is linked to many tumors and various of pathways which could be the potential mechanism for tumor formation. Given the important tumor-promoting role of PTTG1 in a variety of tumors, it is necessary to comprehensively understand the potential of PTTG1 as a potential therapeutic target in different tumors through pan-cancer analysis. The aim of this study was to perform a preliminary analysis of PTTG1 expression, mutational information, prognostic value and potential tumor regulatory mechanisms by integrating tumor genome data from multiple public databases.

Specifically, in the present study, the analysis of gene alteration, PTTG1 expression, prognostic function, and PTTG1-related immune analysis in 33 types of tumors was performed based on various databases such as The Cancer Genome Atlas (TCGA) database, the Genotype-Tissue Expression (GTEx) database, and the Human Protein Atlas (HPA) database. Additionally, PTTG1-related gene enrichment was analyzed to investigate the potential relationship and possible molecular mechanisms between PTTG1 and tumors.

## Materials and Methods

### Gene Alteration Analysis

cBioPortal (https://www.cbioportal.org/) is a user-friendly tool that can be used to analyze genetic alterations in different tumors based on public cohort. In this study, the “TCGA Pan Cancer Atlas Studies” was chosen, the name of the target gene “PTTG1” was typed in the input box to query the summary of genetic alterations, including the types of alteration and copy number data. In addition, the mutation site data could be obtained under the “mutation” section of cBioPortal.

### The Gene Expression at Transcription and Translation Level

TIMER2.0 tool (http://timer.cistrome.org/) is an easy-to-use online tool that can be used to perform a systemic and comprehensive analysis of gene expression, relation between different genes, and immune-related analysis including the cancer-associated fibroblasts (CAF) and other immune cell infiltration in various types of tumors. In the present study, the analysis of PTTG1 expression at the transcription level in various tumors compared to corresponding normal tissues was analyzed based on the TCGA database. Since the normal tissue was limited in some types of tumors, supplementary analysis for these tumors was performed using the GEPIA2 tool (http://gepia2.cancer-pku.cn/#analysis). Using the “Expression Analysis” module of GEPIA2, the expression of PTTG1 in tumors and corresponding normal tissues was obtained according to the TCGA and GTEx datasets.

The Human Protein Atlas (HPA) (https://www.proteinatlas.org/) dataset is a high-quality database that provides researchers with staining-based expression and RNA sequence data. PTTG1 expression at the translation level could be obtained under the “Pathology” and “Tissue” modules. Additionally, an RNA expression overview could also be obtained from the HPA database.

### Survival Analysis

The overall survival (OS) and disease-free survival (DFS) of all involved cancers based on the TCGA dataset could be analyzed using GEPIA2. In this study, the “survival analysis” section of GEPIA2 was used, and the patients were split into two groups at 50–50% cutoff based on PTTG1 expression. Then, the survival map and corresponding Kaplan-Meier curves were obtained together with the *p* value and hazard ratio (HR). The Kaplan-Meier plotter tool (http://kmplot.com/analysis/) was used to harvest the OS, DFS, post-progression survival (PPS), distant metastasis-free survival (DMFS), and progression-free survival (PFS) according to the Gene Expression Omnibus (GEO) database as supplement analysis. “Autoselect best cutoff” was set in Kaplan-Meier plotter tool and the patient would be separate into 2 separate groups to obtain the survival curves. The log-rank *p* value, HR, and 95% confidence intervals were also calculated.

### Construction of Related Gene Network

The STRING tool (https://string-db.org/) is a website tool used to explore protein-protein interactions (PPIs), which are already known or predicted. The PPI network of PTTG1 was constructed using the STRING tool. We identified 39 proteins that directly interacted with PTTG1, and 39 proteins were identified through experiments. Additionally, the GEPIA2 tool was employed to acquire the top 100 genes related to PTTG1 based on all tumor data from the TCGA cohort. The top five genes correlated with PTTG1 were selected to explore the correlation between them and PTTG1. The “Correlation Analysis” section of GEPIA2 was used to obtain the *p* values, correlation coefficient values and dot plots. Spearman’s correlation tests were also applied to these five genes using the TIMER2.0 tool. After a cross-analyzed between PTTG1-interacted genes and PTTG1-correlated genes, a Venn diagram was acquired. GeneMANIA (https://genemania.org/) was used to identify potential PTTG1 related genes.

### KEGG Pathway and GO Enrichment

Based on the combination of PTTG1-interacted genes and PTTG1-correlated genes the R package “clusterProfifiler” was employed to achieve the Kyoto Encyclopedia of Genes and Genomes (KEGG) pathway analysis and Geno Oncology (GO) enrichment analysis including biological process, cellular component, and molecular function.

### Immune-Related Analysis

The TIMER2.0 tool was also employed to investigate the correlation between PTTG1 and cancer-associated fibroblasts (CAFs) across diverse tumors based on the TCGA dataset. The “cancer-associated fibroblasts” was chosen in the immune section and the “PTTG1” was input into the Gene expression box. Then, the CAF heat map and corresponding dot plots were obtained.

The Sangerbox tool (http://sangerbox.com/Tool) is a powerful tool that can be used to perform many biological information analyses. In this study, the sangerbox was used to determine the relationship between PTTG1 expression and immune checkpoint, microsatellite instability (MSI), and tumor mutational burden (TMB).

The UCSC website (https://xenabrowser.net/) was used to obtain the pan-cancer data, which included 11,060 samples. In addition, the expression of PTTG1 was analyzed based on the samples after normalization and log2 (x+0.001) transformed. In addition, we selected the gene expression profiles of each tumor and mapped the profiles to GeneSymbol. The R package ESTIMATE was used to calculate the StromalScore, ImmuneScore, and ESTIMATEScore for each tumor (Yoshihara et al., 2013).

## Results

### The Involved Tumor Types and Genetic Alteration Analysis Data

**FIGURE 1:**
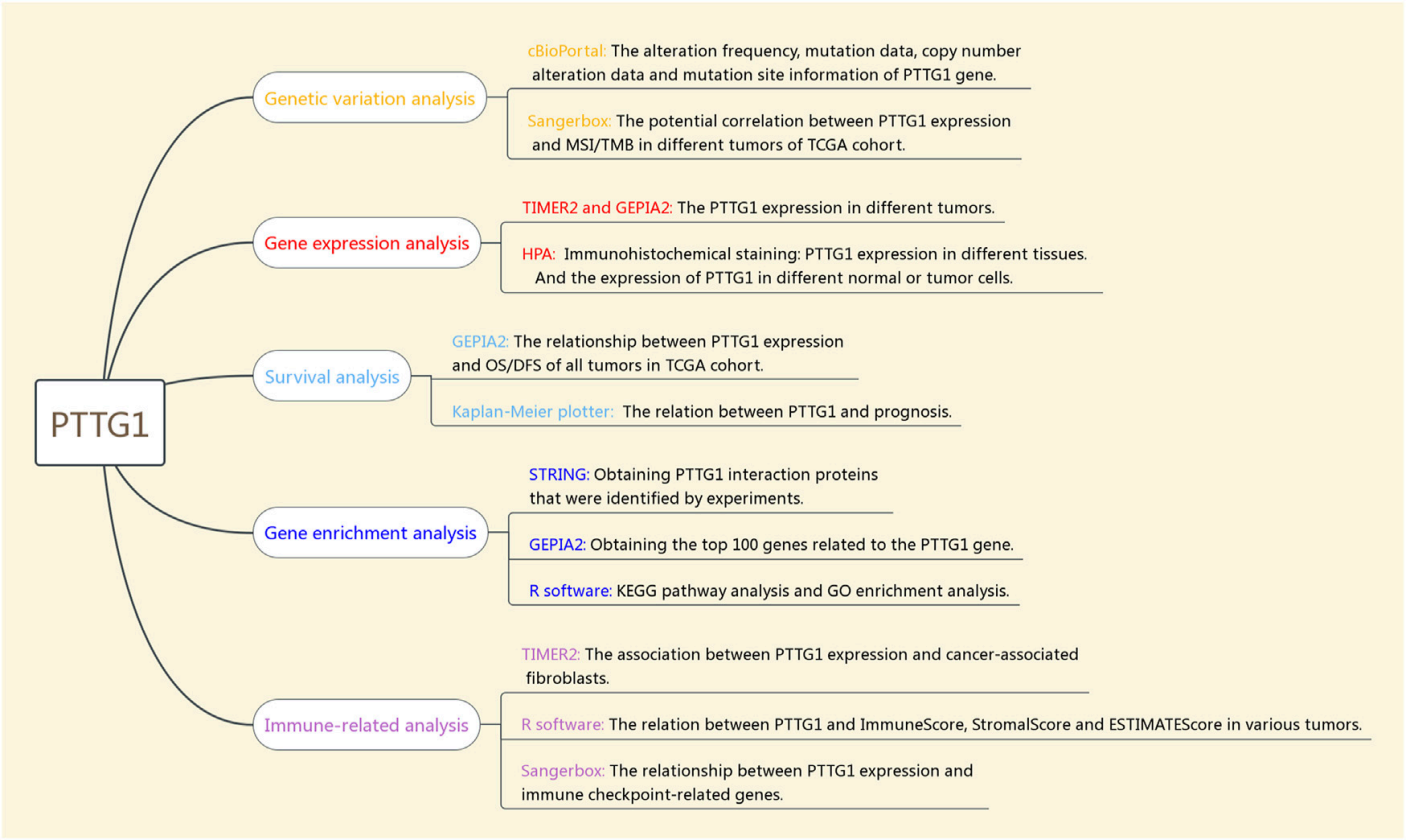
Setup of the integrative and comprehensive Pan-cancer Analysis of PTTG1.

**FIGURE 2:**
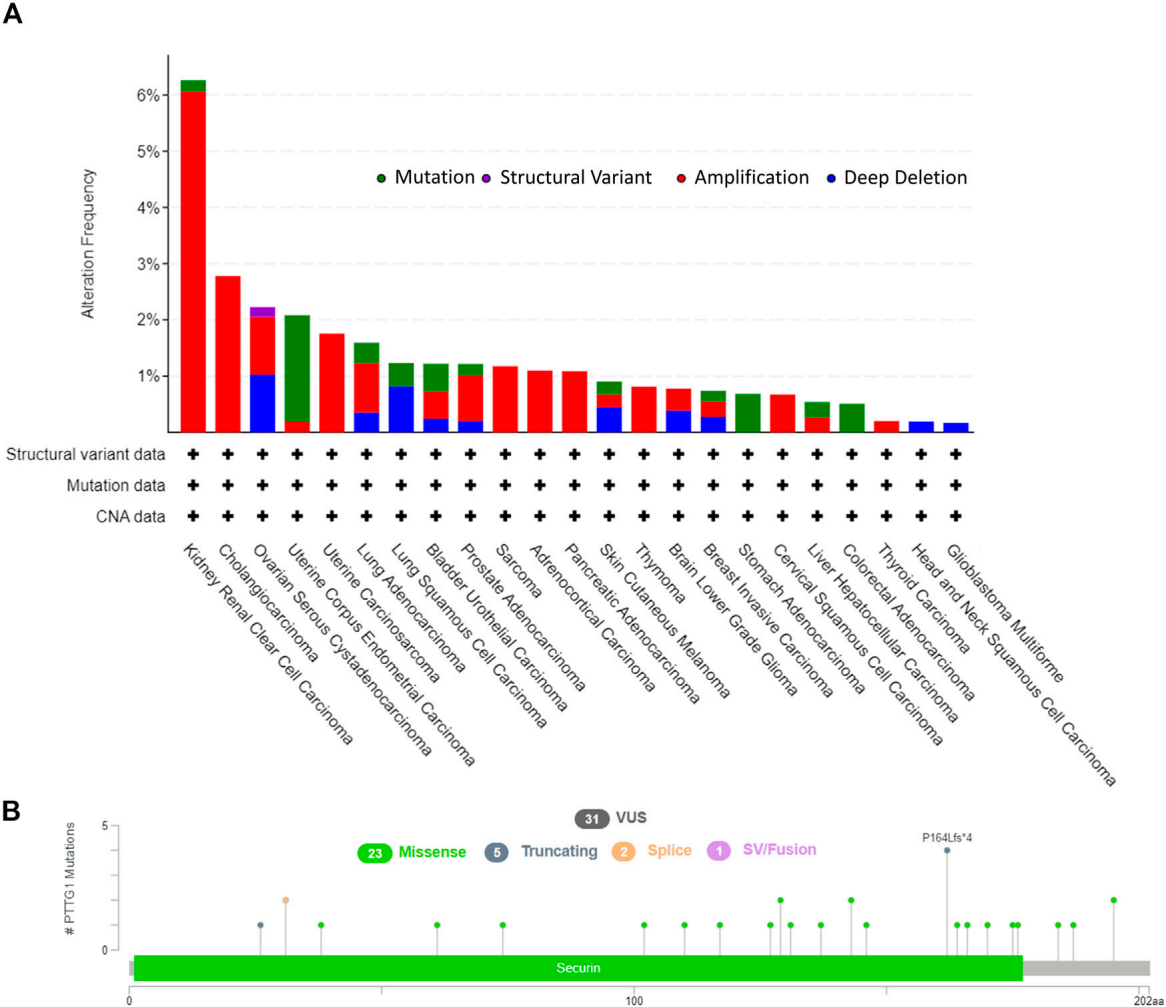
The genetic alteration of PTTG1 in various of tumors. **(A)** The alteration frequency with corelative type was displayed for each tumor. **(B)** The mutation types, sites and case number of PTTG1 genetic alteration were displayed. VUS: variant of uncertain (or unknown) significance.

The tumors that were involved in this study and the corresponding abbreviations were shown in Supplementary Table S1. The workflow of the steps for the present study was shown in Figure 1. And the number of patients from TCGA were shown in Supplementary Table S2. cBioPortal was used to obtain the PTTG1 alteration type and the corresponding frequency. The alteration frequency was 6.26% in kidney renal clear cell carcinoma (KIRC), and most of the frequency was amplified at 6.06% compared to the mutation at 0.2%. Of note, the amplification was the only alteration type in cholangiocarcinoma, sarcoma (SARC), adrenocortical carcinoma (ACC), pancreatic adenocarcinoma (PAAD), thymoma (THYM), cervical squamous cell carcinoma, and thyroid carcinoma (Figure 2A). In addition, mutation types, together with the site of PTTG1, were also achieved (Figure 2B). There are 31 variants of uncertain/unknown significance (VUS) in PTTG1. Missense mutations were the most common mutations in PTTG1, followed by truncating, splice, and fusion.

### The Gene Expression Results

First, PTTG1 expression in normal tissues, single cells, and various tumor tissues was analyzed based on a consensus database (Supplementary Figure S1). The consensus database is a high-quality database that includes many other databases, such as HPA, GTEx, and FANTOM five cohorts. This shows that PTTG1 expression was enhanced in the bone marrow, lymphoid tissue, and testis with normal tissue specificity (Supplementary Figure S1A). Additionally, PTTG1 was overexpressed in some cell types, such as early spermatids and spermatocytes, with RNA single cell type specificity (Supplementary Figure S1B). However, the expression of PTTG1 showed low cancer specificity in the RNA cancer category (Supplementary Figure S1C). Therefore, PTTG1 expression was at the same level among different cancer categories, but PTTG1 may be overexpressed in some normal tissues or cells compared to other normal tissues.

**FIGURE 3:**
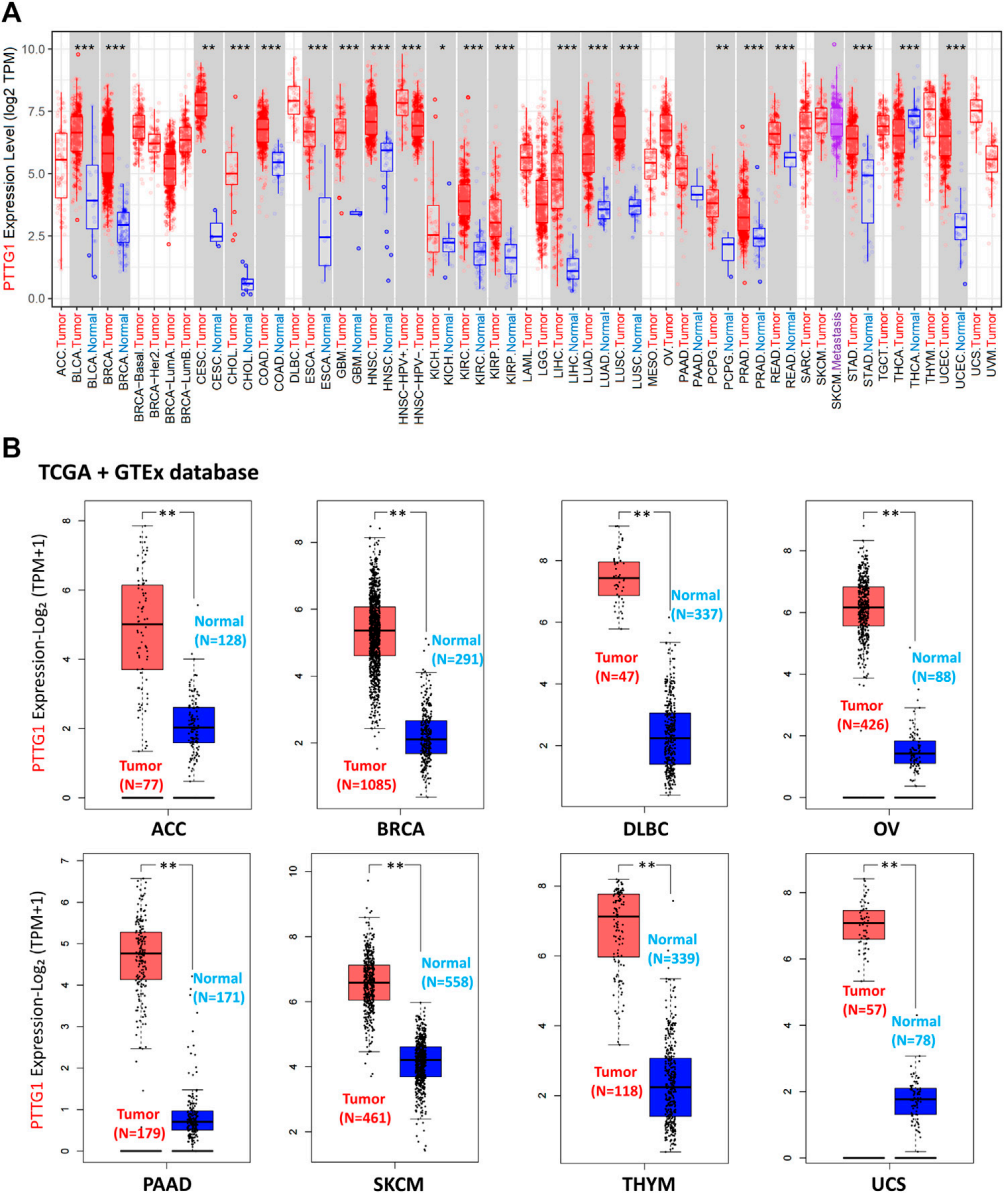
The expression of PTTG1 in different type of tumors at transcription level. **(A)** The PTTG1 expression in different tumors based on TCGA database. **(B)** The expression level of PTTG1 in various type of tumors based on the combination of TCGA and GTEx cohort. *p < 0.05, **p < 0.01, ***p < 0.001.

Using the TIMER2.0 tool, the expression of PTTG1 in diverse tumors and corresponding normal tissues was obtained based on the TCGA database. PTTG1 expression in bladder urothelial carcinoma (BLCA), breast invasive carcinoma (BRCA), cervical squamous cell carcinoma and endocervical adenocarcinoma (CESC), cholangiocarcinoma (CHOL), colon adenocarcinoma (COAD), esophageal carcinoma (ESCA), glioblastoma multiforme (GBM), head and neck squamous cell carcinoma (HNSC), kidney chromophobe (KICH), kidney renal clear cell carcinoma (KIRC), kidney renal papillary cell carcinoma (KIRP), liver hepatocellular carcinoma (LIHC), lung adenocarcinoma (LUAD), lung squamous cell carcinoma (LUSC), pheochromocytoma and paraganglioma (PCPG), prostate adenocarcinoma (PRAD), rectum adenocarcinoma (READ), stomach adenocarcinoma (STAD), and uterine corpus endometrial carcinoma (UCEC) were higher than those in normal tissues (Figure 3A). However, it could be found that the PTTG1 expression was lower in Thyroid carcinoma (THCA) than the normal tissue (*p* < 0.001).

As the normal samples in some tumor, such as BRCA and PAAD, were not enough to analyze, we did the supplementary analysis. Using the GEPIA2 tool, the supplementary analysis of PTTG1 expression between tumor and normal tissues depended on the combination of TCGA and GTEx datasets was achieved (Figure 3B and Supplementary Figure S2). PTTG1 expression in ACC, BRCA, lymphoid neoplasm diffuse large B-cell lymphoma (DLBC), ovarian serous cystadenocarcinoma (OV), PAAD, skin cutaneous melanoma (SKCM), THYM, uterine carcinosarcoma (UCS), and sarcoma (SARC) was higher than that in normal tissues (all *p* < 0.01) (Figure 3B and Supplementary Figure S2). However, PTTG1 expression in acute myeloid leukemia (LAML) and testicular germ cell tumors (TGCT) was lower than that in normal tissues (all *p* < 0.01) (Supplementary Figure S2).

**FIGURE 4:**
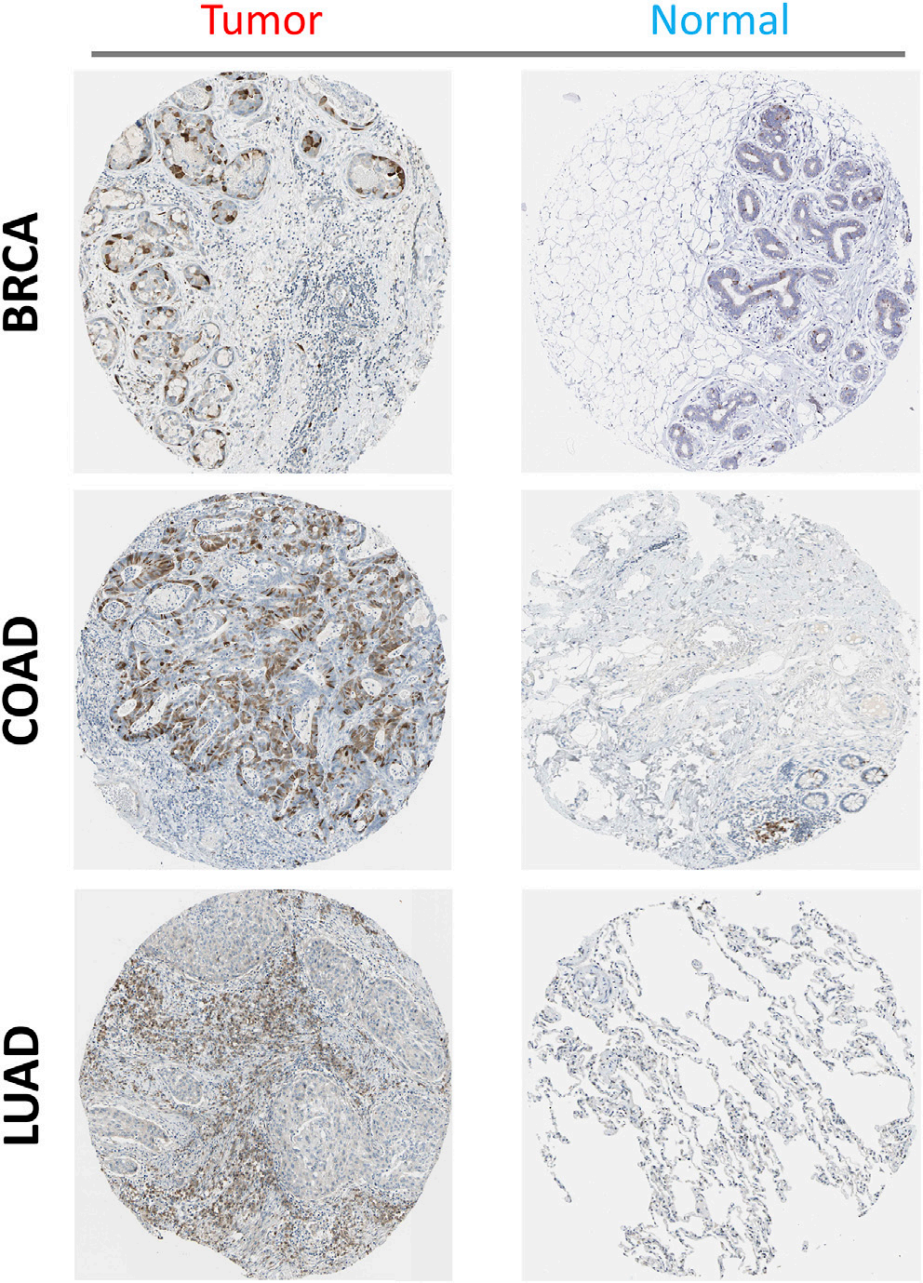
The expression of PTTG1 in different tumors at translation level: three types of tumors were shown (BRCA, COAD and LUAD) compared to the corelating normal tissue (Breast, Colon and Lung).

Additionally, using the HPA database, immunohistochemical staining of PTTG1 in different tumors and normal tissues was performed. PTTG1 expression at the protein level was higher in various tumors, including BRCA, COAD, and lung adenocarcinoma (LUAD) (Figure 4).

### Survival Analysis

**FIGURE 5:**
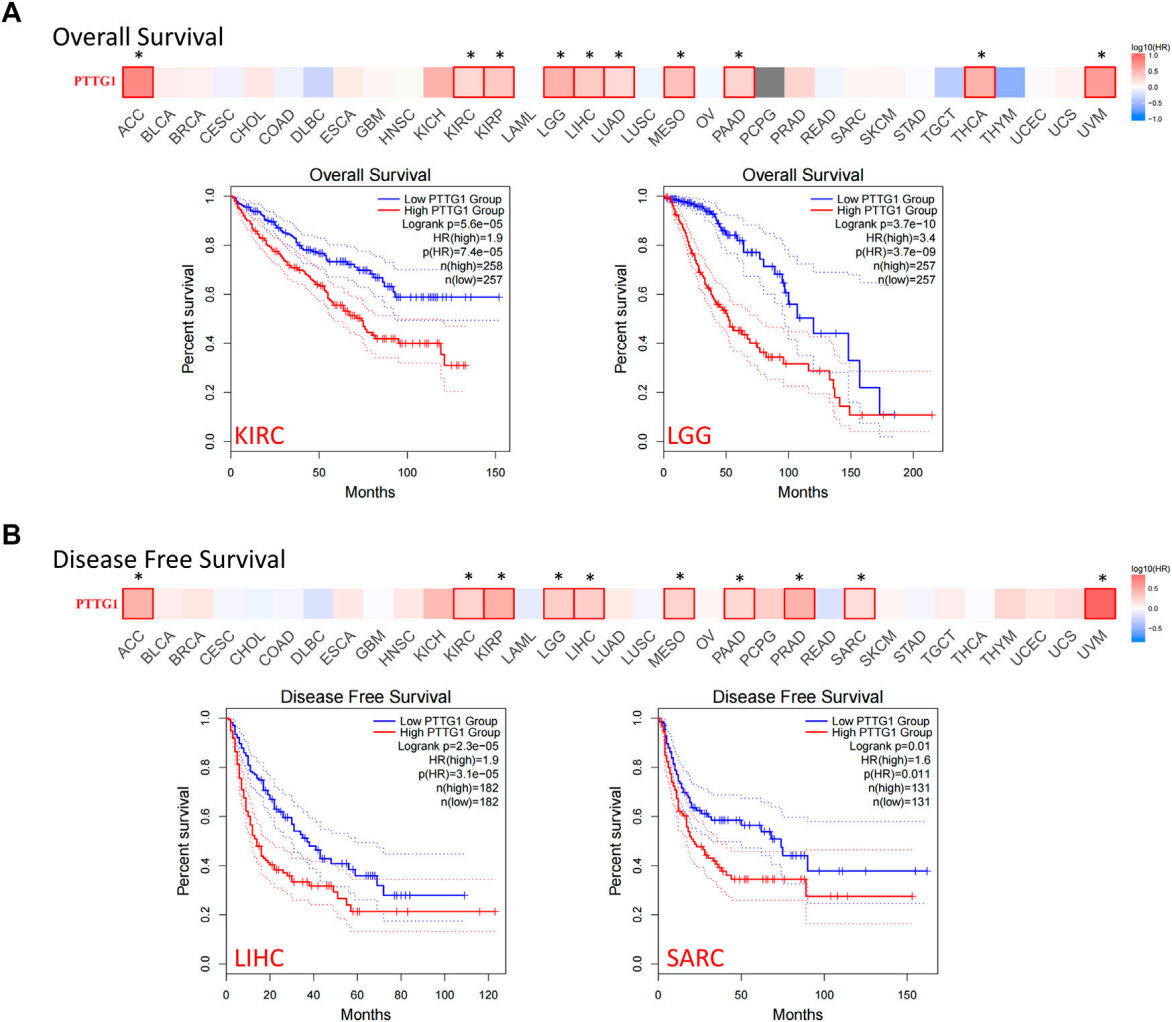
The correlation between PTTG1 expression and survival prognosis of different tumor based on TCGA database. **(A)** The correlation between expression and overall survival (OS) in different tumors. **(B)** The correlation between PTTG1 expression and disease-free survival (DFS) in different tumors. The survival maps and Kaplan–Meier curves of two typical tumors were also shown. Dotted lines: 95% confidence interval. *p < 0.05.

The data from TCGA database were separated into two groups: the 50% higher expression of PTTG1 group and the 50% lower expression of PTTG1 group. The correlation between the expression of PTTG1 and prognosis across different types of tumors was analyzed. Patients with high expression of PTTG1 were associated with poor overall survival (OS) in ACC, KIRC, KIRP, lower grade glioma (LGG), LIHC, LUAD, mesothelioma (MESO), PAAD, THCA, and uveal melanoma (UVM) (Figure 5A, all *p* < 0.05). Among these types of tumors, the Kaplan–Meier curves of KIRC and LGG are shown. Patients with high expression of PTTG1 were associated with poor disease-free survival (DFS) in the ACC, KIRC, KIRP, LGG, LIHC, MESO, PAAD, PRAD, SARC, and UVM (Figure 5B). The Kaplan–Meier curves of LIHC and SARC are shown.

However, no significance could be achieved in other types of tumors if we separated PTTG1 expression as a 50–50% cutoff. Therefore, using the Kaplan–Meier Plotter tool, a further analysis was performed by setting the cutoff as “Autoselect best cutoff”. The high expression of PTTG1 was linked to poor prognosis in BLCA, BRCA, CESC, and STAD (Supplementary Figure S3). It indicated that PTTG1 may be involved in the malignant progression of a variety of tumors and may serve as a prognostic biomarker.

### Gene Enrichment Analysis

**FIGURE 6:**
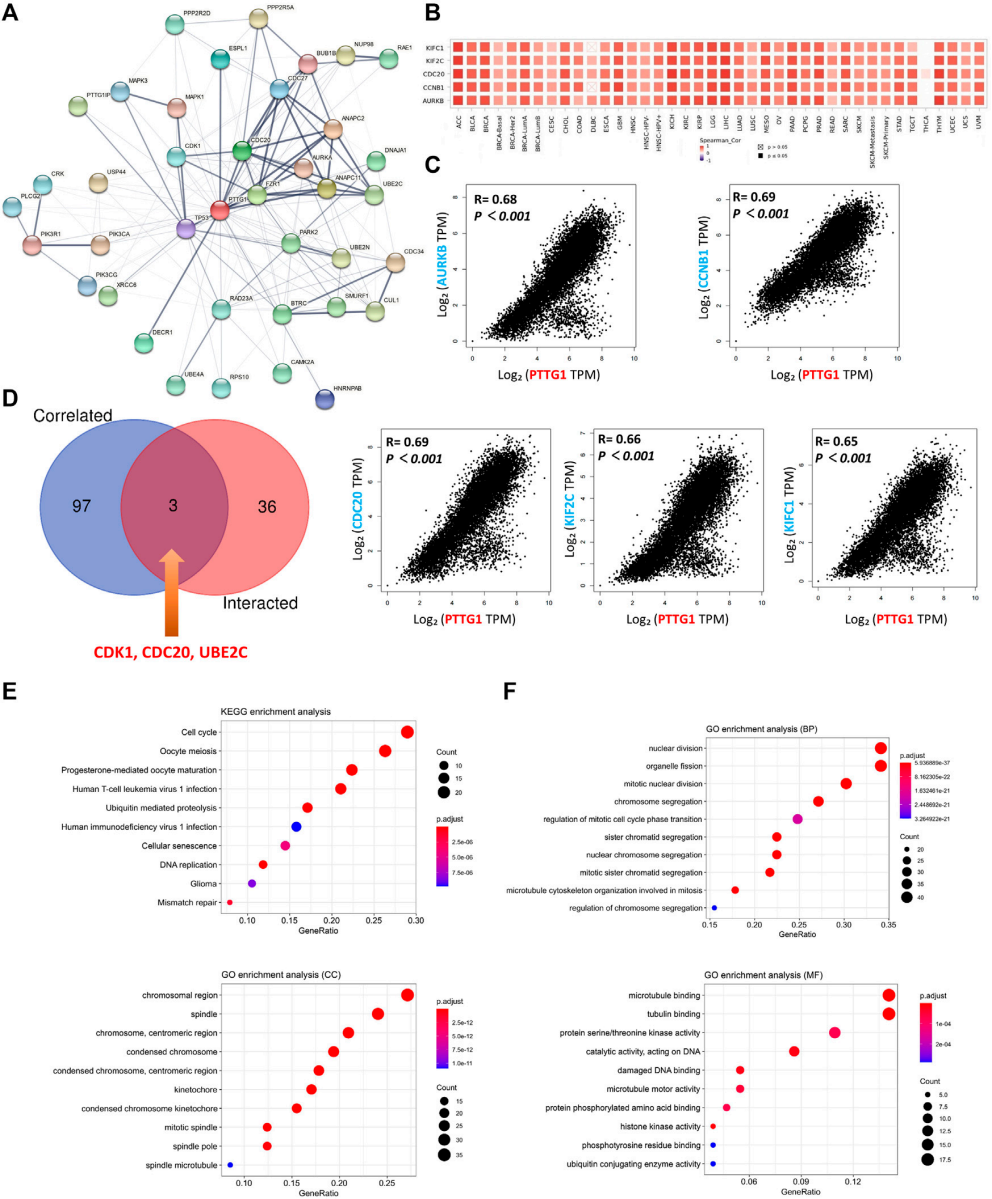
The related gene analysis for PTTG1. **(A)** A protein-protein interaction (PPI) network of 39 PTTG1-interacted experimentally verified proteins were shown. **(B)** The top 100 genes which were linked to the PTTG1 expression were obtained, and the top five genes was shown, named AURKB, CCNB1, CDC20, KIF2C and KIFC1. The heatmap data which displayed the correlation of the top five genes with different tumors were shown. **(C)** The expression correlations between the top five genes and PTTG1 were also displayed. **(D)** The intersection analysis of the PTTG1-correlated and PTTG1-interacted genes was conducted and three genes were obtained, namely CDK1, CDC20 and UBE2C. **(E)** KEGG pathway analysis was obtained according to PTTG1-correlated and interacted genes; **(F)** The GO enrichment in biological process (BP) terms, cellular component (CC) terms, and molecular function (MF) terms.

To further explore the potential mechanism of PTTG1 in tumor development and adverse clinical outcomes, the protein-protein interaction (PPI) network analysis was performed and PTTG1-interacted and PTTG1-correlated genes were extracted. The PPI network and potential related gene network analyzed by GeneMANIA are shown in Figure 6A and Supplementary Figure S4. Using the STRING and GEPIA2 tools, 39 PTTG1 interacted genes and 100 PTTG1 correlated genes were identified (Supplementary Table S3). The top five PTTG1-correlated genes were kinesin family member C1 (KIFC1), kinesin family member 2C (KIF2C), cell division cycle 20 (CDC20), cyclin B1(CCNB1), and aurora kinase B (AURKB). These five genes were analyzed to explore the relationship between their expression and PTTG1 expression in each type of tumors (Figure 6B). Additionally, the relationship between their expression and PTTG1 expression across various tumors was also analyzed (Figure 6C). After the cross analysis between the interacting and correlated genes of PTTG1, three genes were identified: cyclin dependent kinase 1 (CDK1), CDC20, and ubiquitin conjugating enzyme E2 C (UBE2C) (Figure 6D).

By using the combination of the PTTG1 interacting and correlated gene sets, the Kyoto Encyclopedia of Genes and Genomes (KEGG) pathway analysis and Geno Oncology (GO) enrichment analysis was performed. KEGG pathway analysis showed that PTTG1 was linked to cell cycle, oocyte meiosis, human immunodeficiency virus 1 (HIV-1) infection, glioma, and mismatch repair (Figure 6E). The GO enrichment analysis showed that PTTG1 was linked to organelle fission, sister chromatid segregation in biological process (BP) terms, chromosome, centromeric region, mitotic spindle in cellular component (CC) terms, protein serine/threonine kinase activity, and damaged DNA binding in molecular function (MF) terms (Figure 6F).

### Immune-Related Analysis

**FIGURE 7:**
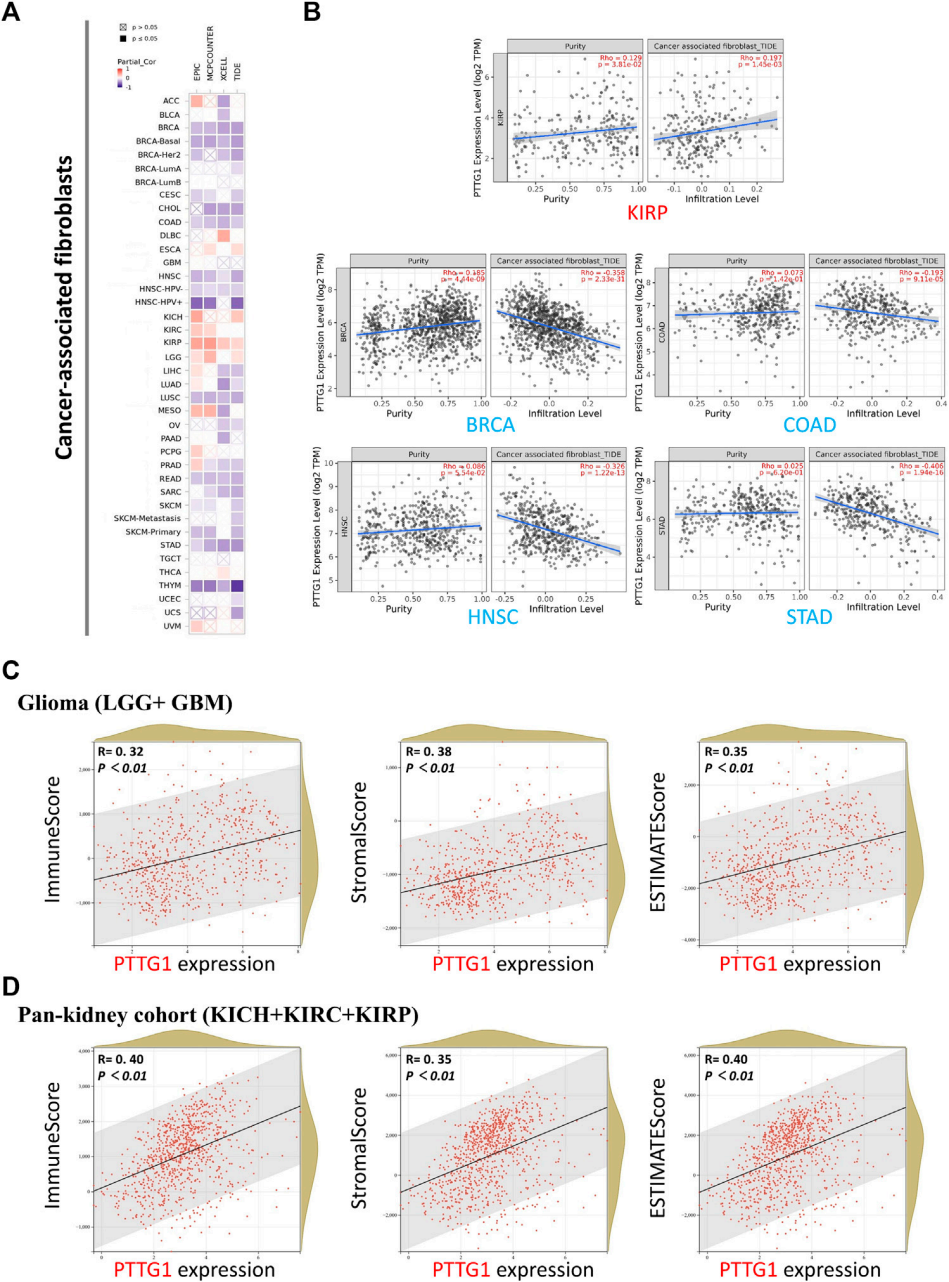
Cancer-associated fibroblasts analysis and immune check point of PTTG1. **(A)** Association between PTTG1 expression and immune infiltration of cancer-associated fibroblasts in different tumors. **(B)** The scatter plots of cancer-associated fibroblasts immune infiltration in different tumors generated based on a certain algorithm. The analysis of correlation between PTTG1 expression and ImmuneScore, StromalScore and ESTIMATEScore was conducted. Two kinds of typical tumors **(C)** glioma (LGG + GBM) **(D)** Pan-kidney cohort (KICH + KIRC + KIRP) were displayed.

Four different algorithms, EPIC, MCPCOUNTER, XCELL, and TIDE were used to analyze the relationship between PTGG1 expression and cancer-associated fibroblasts (CAFs). The same tendency could be obtained in most of the algorithms, which would be viewed as reliable. The expression of PTTG1 was positively related to CAF infiltration in KIRP and negatively related to CAF infiltration in various tumors such as BRCA, CESC, and COAD (Figure 7A). Typical scatter plots developed by the algorithm are shown in Figure 7B. The R package “psych” was used to analyze the relation between PTTG1 and immune infiltration score. Significant differences were observed in most of the involved tumors in all the ImmuneScore, StromalScore, and ESTIMATEScore (Supplementary Table S4). PTTG1 was positively correlated with all the ImmuneScore, StromalScore, and ESTIMATEScore in glioma (GBM + LGG) and pan-kidney cohorts (KICH + KIRC + KIRP) (Figures 7C,D, all *p* < 0.05).

**FIGURE 8:**
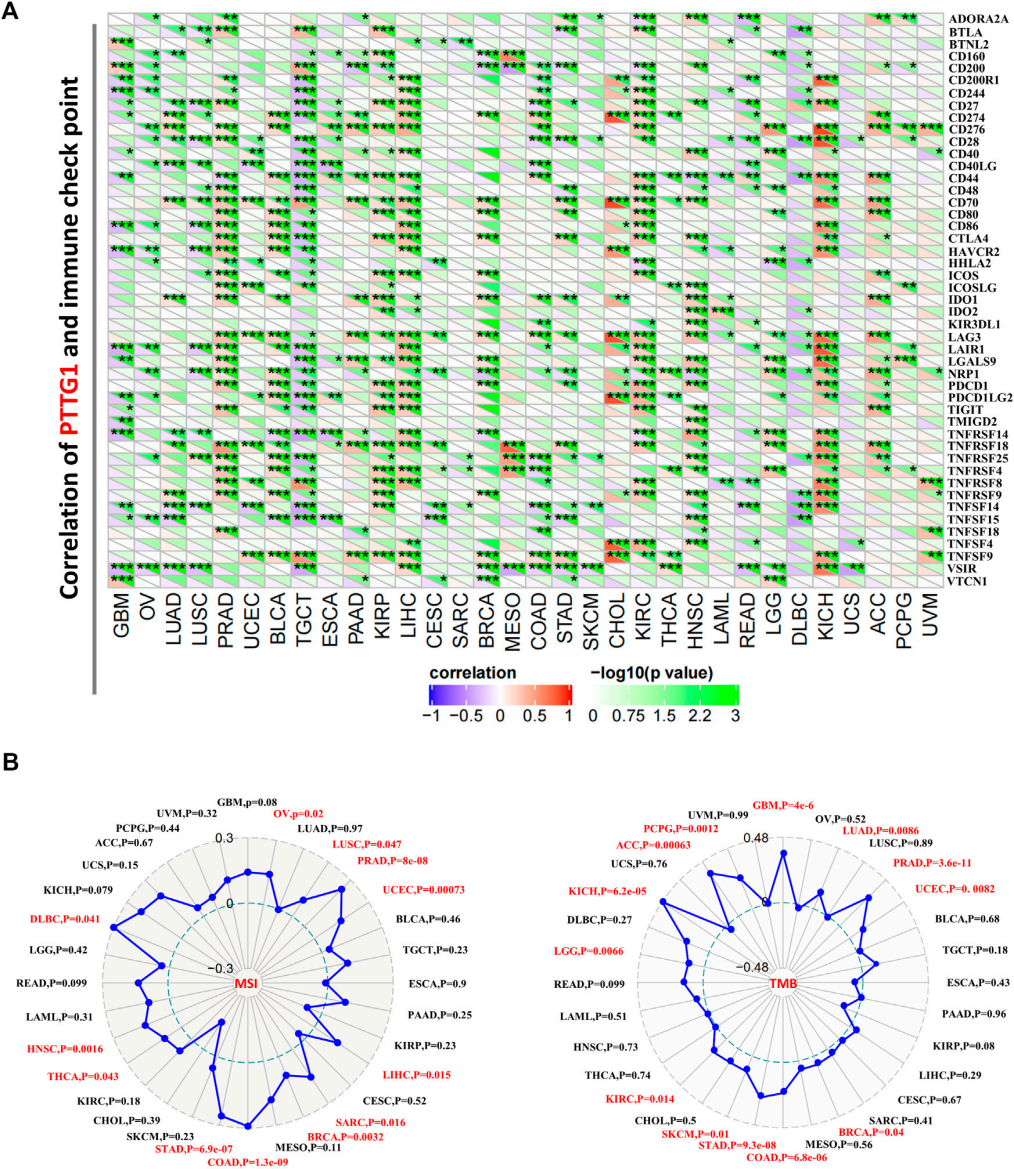
**(A)** Association between PTTG1 expression and immune checkpoint genes expression in different tumors. **(B)** Correlation between PTTG1 expression and microsatellite instability (MSI) or tumor mutational burden (TMB). *p < 0.05; **p < 0.01; ***p < 0.001.

Immune checkpoint analysis showed that PTTG1 was negatively correlated with most of the checkpoint genes in TGCT, while PTTG1 was positively correlated with immune checkpoints in some other tumors such as PRAD, KIRP, and KIRC (Figure 8A, all *p* < 0.05). In addition, the correlation between the expression of PTTG1 and MSI/TMB was also analyzed. PTTG1 expression was positively correlated with MSI in OV, LUSC, PRAD, UCEC, LIHC, SARC, BRCA, COAD, STAD, HNSC, and DLBC (Figure 8B, all *p* < 0.05). PTTG1 expression was positively correlated with TMB in GBM, LUAD, PRAD, UCEC, BRCA, COAD, STAD, SKCM, KIRC, LGG, KICH, ACC, and PCPG (Figure 8B, all *p* < 0.05).

## Discussion

PTTG1 is a crucial gene involved in mitosis, especially during sister chromatid separation (Hatcher et al., 2014). Various research has shown that PTTG1 is linked to tumor formation and prognosis in numerous tumors, such as glioma, KIRC, and LIHC (Wondergem et al., 2012; Lin et al., 2019a; Zhi et al., 2019). But, however, the analysis between expression of PTTG1 and human pan-cancer was still poor. In the present study, using various tools such as TIMER2.0, GEPIA2, HPA, and sangerbox, a comprehensive analysis of PTTG1 was achieved based on the data from TCGA, GEO, and GTEx cohorts. The data were used to explore PTTG1 genetic alterations, gene expression, survival, and immune-related infiltration. In addition, gene enrichment was used to obtain a deeper insight into the potential mechanism of PTTG1 in numerous tumors. Hence, the relation and potential mechanism between PTTG1 and human pan-cancer could be obtained, which would also provide a novel approach for tumor treatment.

Genetic mutation analysis showed that the altered rate was more than 6% in KIRC followed by 2.78% in CHOL (Figure 2). In recent years, growing evidence has indicated that mutated-gene-target therapy is a promising approach for tumor therapy (Duffy et al., 2017; Synnott et al., 2017; Kaur et al., 2018). Thus, the PTTG1-tageted gene could also be a potential treatment for tumors such as KIRC and CHOL. PTTG1 expression analysis showed that the expression of PTTG1 was overexpressed in most of the involved tumors, such as BLCA, BRCA, and OV, based on TCGA and GTEx cohorts (Figure 3). Hence, our results further support previous studies showing that PTTG1 is an oncogene (Yoon et al., 2012; Noll et al., 2015). Interestingly, the expression of PTTG1 was lower in cancer than corresponding normal tissue in LAML and TGCT (Supplementary Figure S2), which might mean the PTTG1 would be a “double-edged sword” for the PTTG1 was oncogene in most of tumors but also tumor suppressor gene in tumors such as LAML and TGCT. Although PTTG1 was lower expression in some tumors, it was always associated with poor prognosis. As the OS was normally viewed as the best index for tumor therapy, the DFS also plays a crucial role in surgical treatment and radiotherapy for it could be used to evaluate the outcome of the treatment. For the tumors such as ACC, KIRC and LGG, the high expression of PTTG1 was linked to both the OS and DFS. Thus, it makes the PTTG1 be a potential biomarker for these tumors in designing the treatment plan. Previous studies have shown that PTTG1 could be a prognostic biomarker in some tumors, and our results further extend their finding (Repo et al., 2017; Kaur et al., 2018; Romero Arenas et al., 2018; Fraune et al., 2020). Additionally, PTTG1 could also affect the drug sensitivity in some tumors such as BRCA, SKCM, and OV, which provided us with a novel treatment idea that the PTTG1-target drug or operation could be applied to the patient to enhance the chemotherapy outcomes (Nakachi et al., 2016; Caporali et al., 2017; Meng et al., 2020).

Using the cross analysis of PTTG1 interacting and correlated genes, three common genes were identified: CDK1, CDC20, and UBE2C (Figure 6D). The combination of PTTG1 and CDC20 has already been found and is considered to be a hub gene in some tumors such as PRAD, KIRC, and BRCA (Dai et al., 2021; Lin et al., 2019b; Deng et al., 2021). In addition, PTTG1, CDK1, and UBE2C are also viewed as hub genes in COAD (Deng et al., 2021). Moreover, the UBE2C-mediated P53 ubiquitination could be a potential target in LIHC therapy (Zhu et al., 2021). Interestingly, the combination of PTTG1 and the other three genes was not only linked to tumors like LIHC but also to acute type A aortic dissection (Luo et al., 2019; Jiang and Si, 2019). Consequently, there could be a potential mechanism between these four genes, which warrants further exploration. The KEGG pathway analysis indicated that PTTG1 was correlated with the cell cycle, DNA replication, and mismatch repair (Figure 6E). This might reveal a potential mechanism between PTTG1 and tumor formation. EMT is a normal process during wound healing but may also contribute to fibrosis and cancer progression. During this process, epithelial cells may lose their polarity and adherence ability and become invasive (Lamouille et al., 2014). Overexpression of PTTG1 may lead to disorders in the process of cell proliferation, DNA replication, and mismatched DNA repair. Then, normal epithelial cells may become tumor cells and invade. The KEGG pathway also showed that PTTG1 was linked to human T-cell lymphotropic virus type-1 (HTLV-1) infection, and HTLV-1 is a retrovirus that is closely related to adult T-cell leukemia/lymphoma (ATL) (Guerrero et al., 2020). The cell cycle is necessary for the proliferation of HTLV-1, and the tax protein encoded by HTLV-1 may damage the mitotic spindle, resulting in incorrect sister chromatid separation (Levy et al., 2020). Consequently, it might be another potential mechanism between PTTG1/HTLV-1 and tumor formation, such as ALT. GO enrichment showed that PTTG1 was associated with mitosis and protein serine/threonine kinase activity (Figure 6F). MAPK, a member of the serine-threonine kinase family, is associated with many tumor processes, such as tumor formation and invasion (Fang and Richardson, 2005; Burotto et al., 2014; Suarez-Carmona et al., 2017; Sun et al., 2019; Lee et al., 2020). Hence, the MAPK pathway might also be a potential mechanism associated with PTTG1 and tumor formation (Noh et al., 2009; Liang et al., 2015; Hu et al., 2019).

CAF has been proven to be closely associated with various tumor procedures and is one of the most important parts of the tumor microenvironment (TME) stroma (Figure 7A) (Chen and Song, 2019; Sahai et al., 2020). Our study showed that PTTG1 was positively correlated with CAF in KIRP and LGG but negatively correlated with diverse tumors such as BRCA, COAD, and STAD. Recent research has indicated that CAF-target therapy could shape the TME (Affo et al., 2017; Kubo et al., 2016). Especially, previous studies have shown that the CAF could contribute to the remodeling of extracellular matrix in LIHC and the exosome could be a potential target to reverse chemoresistance (Zhang et al., 2020a; Zhang et al., 2020b). Consequently, CAF-target therapy with exosome could be a promising approach for tumor such as LIHC. Our results provide insight into CAF in various tumors, which could be helpful for further research in CAF and TME. It would be revolutionary changes that researchers have gained deep insight into the immune checkpoint in recent years (Topalian et al., 2016; Abril-Rodriguez and Ribas, 2017; Mezquita et al., 2018). Immune checkpoint inhibitors for cytotoxic T lymphocyte-associated protein-4 (CTLA-4) and programmed cell death protein-1 (PD-1, also known as PDCD1) have achieved great success (Rotte, 2019; Shukla et al., 2018). Our findings revealed that PTTG1 is positively correlated with both CTLA-4 and PD-1 in some tumors such as ACC, KICH, and HNSC, which may indicate that PTTG1 could be a novel biomarker for PD-1 in these tumors (Figure 8A). Immune infiltration analysis showed that PTTG1 was positively correlated with ImmuneScore, StromalScore, and ESTIMATEScore in tumors such as glioma and kidney tumors. This means that the overexpression of PTTG1 is linked to high infiltration of immune cells and stromal cells but low tumor purity in these tumors (Figure 7 and Supplementary Table S4). Growing evidence has shown that TMB could be a possible biomarker for tumor sensitivity to immune checkpoint blockade (Chalmers et al., 2017; Chan et al., 2019). The microsatellite instability (MSI) and TMB of PTTG1 could be potential biomarkers for immune checkpoint inhibitors, such as PD-1 or CTLA-4. The PD-1 and CTLA-4 is now regarded as promising target in tumor treatment. Thus, the combination of MSI and TMB would be a novel approach to predict the outcome of treatment for tumors (Goodman et al., 2019; Luchini et al., 2019; Schrock et al., 2019; Jang et al., 2020). In the present study, PTTG1 was positively correlated with TMB, MSI, PD-1, and CTLA-4 in tumors such as PRAD, BRCA, and STAD. Consequently, immune checkpoint inhibitors may have a good outcome in these tumors. However, there are some limitations in this study. The data of RNA sequence and immunohistochemical staining was used to analyze the potential role of PTTG1 in human pan-cancer but the analysis of multi-omics is absence. Previous studies showed that multi-omics analysis including RNA sequencing, proteomic and metabolomic analysis could get a good outcome (Su et al., 2021). Consequently, it would be a promising method to analyze the role of PTTG1 in tumor using the multi-omics approach.

The present study shows the results of the comprehensive pan-cancer analysis data of PTTG1. And it is the first analysis that combined the expression of PTTG1, prognosis, KEGG pathway and GO enrichment analysis, immune-related analysis, immune checkpoint, TMB, and MSI. This study provides novel insights into the potential role of PTTG1 in tumor formation and prognostic function.

## Conclusion

In summary, our findings show that PTTG1 acts as an oncogene in most of the involved tumors. Overexpression of PTTG1 may lead to a poor prognosis in various tumors. Additionally, PTTG1 is related to immune-related infiltration, immune checkpoints, TMB, and MSI. Consequently, we identified that PTTG1 could be a prognostic biomarker and a novel target for some tumors.

## Data Availability

The original contributions presented in the study are included in the article/Supplementary Material, further inquiries can be directed to the corresponding author.
