# Supplementary material for: A Comprehensive Pan-Cancer Analysis for Pituitary Tumor-Transforming Gene 1
Source: Front Genet. 2022 Feb 25;13:843579. doi: 10.3389/fgene.2022.843579 (PMC8916819; doi:10.3389/fgene.2022.843579)
Supplement: Supplementary file 4 [file DataSheet1.docx]

Supplementary Material

## Supplementary Figures


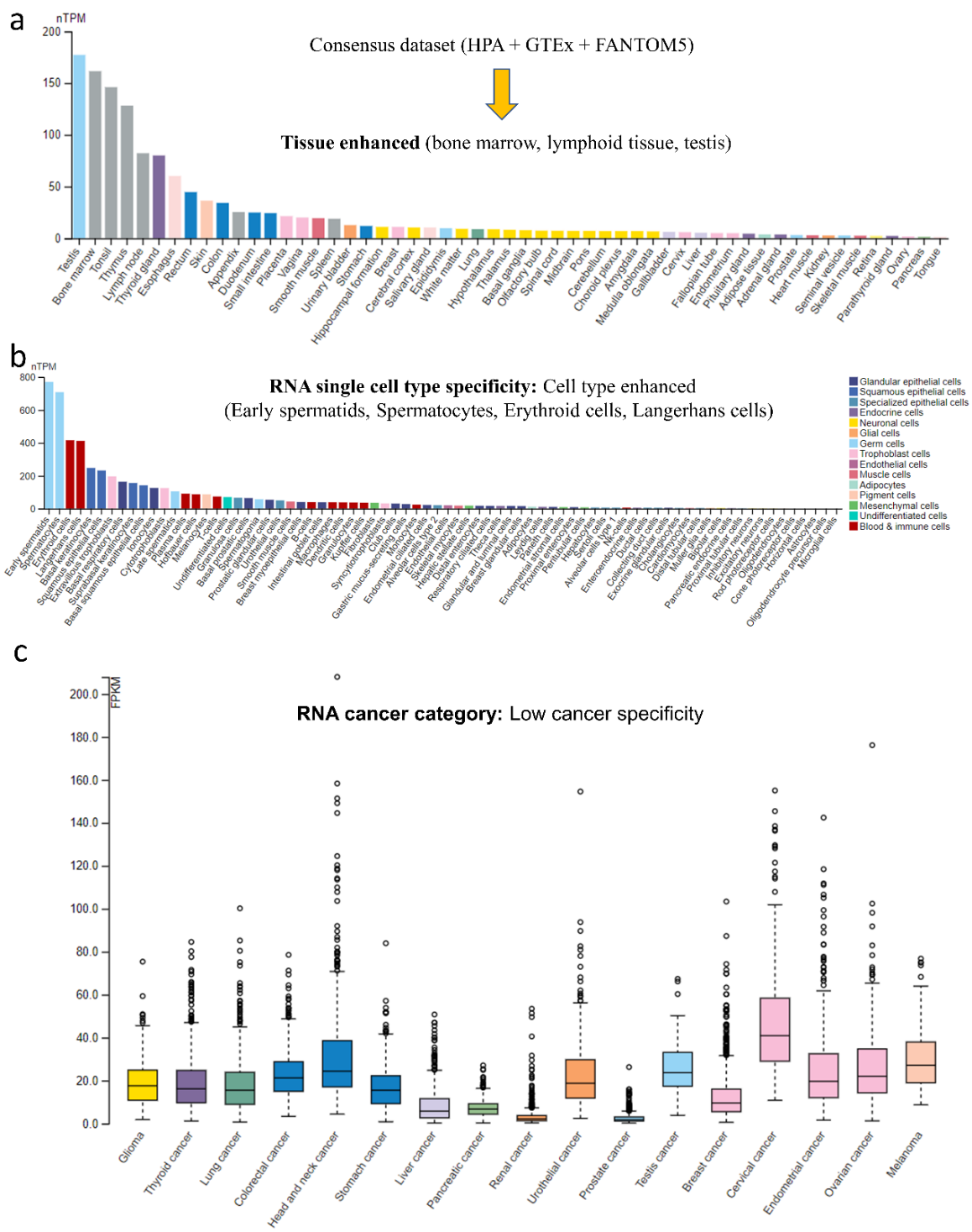


**Supplementary Figure S1.** Expression level of PTTG1 in different normal tissues, single cells and tumor tissues. **(a)** PTTG1 gene in different normal tissues using the consensus datasets of HPA, GTEx and FANTOM5. **(b)** PTTG1 gene in different single cells. **(c)** PTTG1 gene in different tumor tissue.


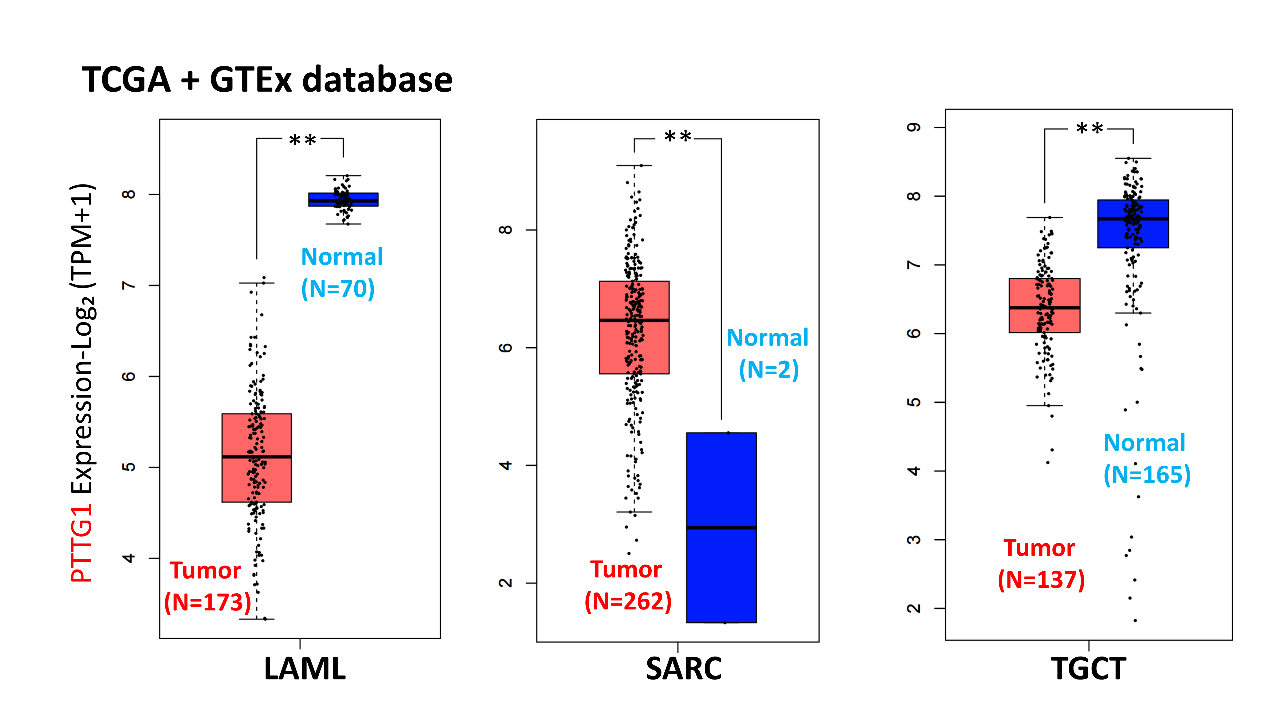


**Supplementary Figure S2.** The expression level of PTTG1 in different type of tumors based on the combination of TCGA and GTEx cohort. ** *p* < 0.01


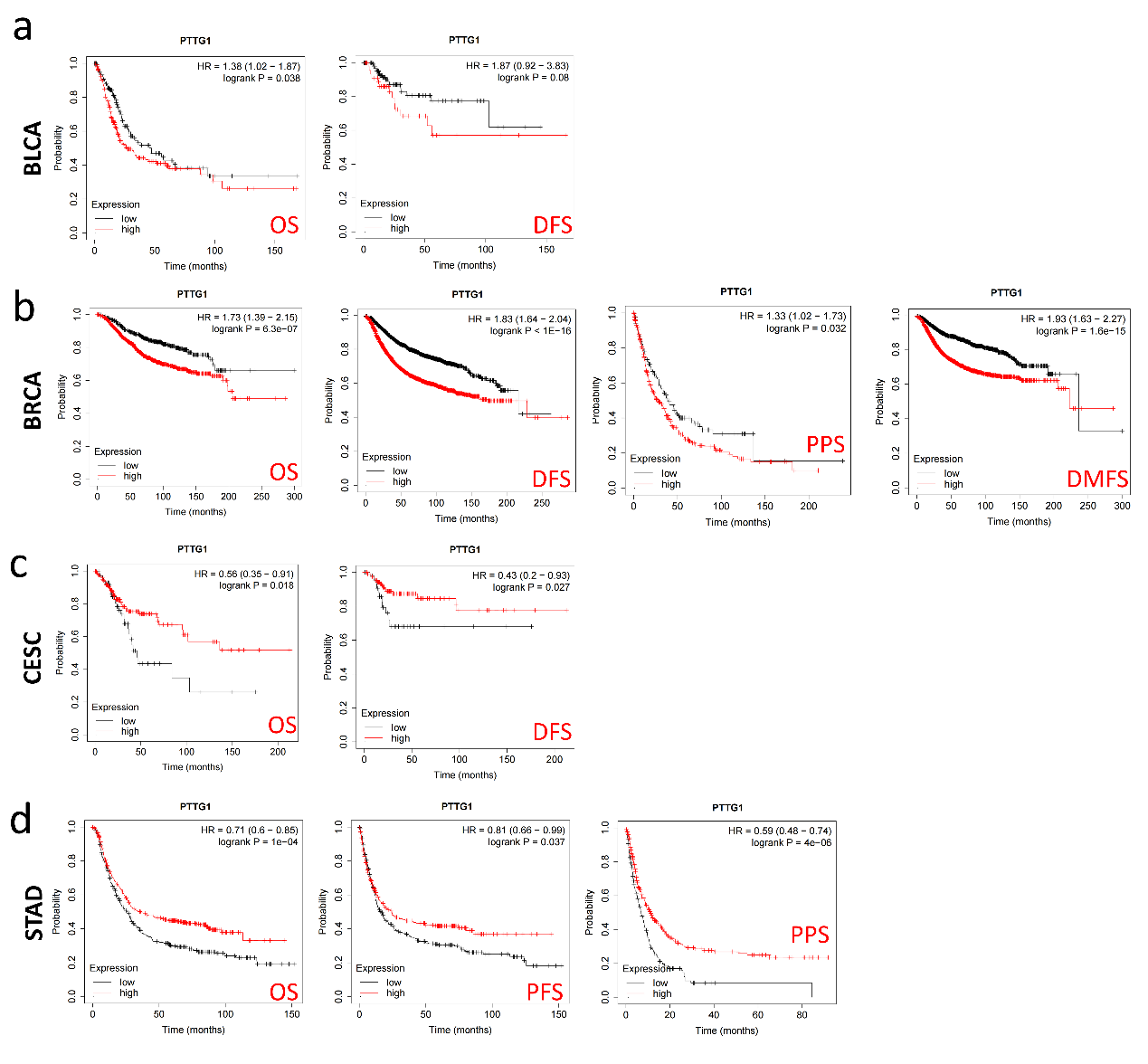


**Supplementary Figure S3.** Correlation between PTTG1 gene expression and prognosis of different tumors based on the Kaplan-Meier plotter. (a) BLCA; (b) BRCA; (c) CESC; (d) STAD. OS overall survival, DFS disease free survival, PPS post progression survival, DMFS Distant Metastasis Free Survival, PFS progression free survival


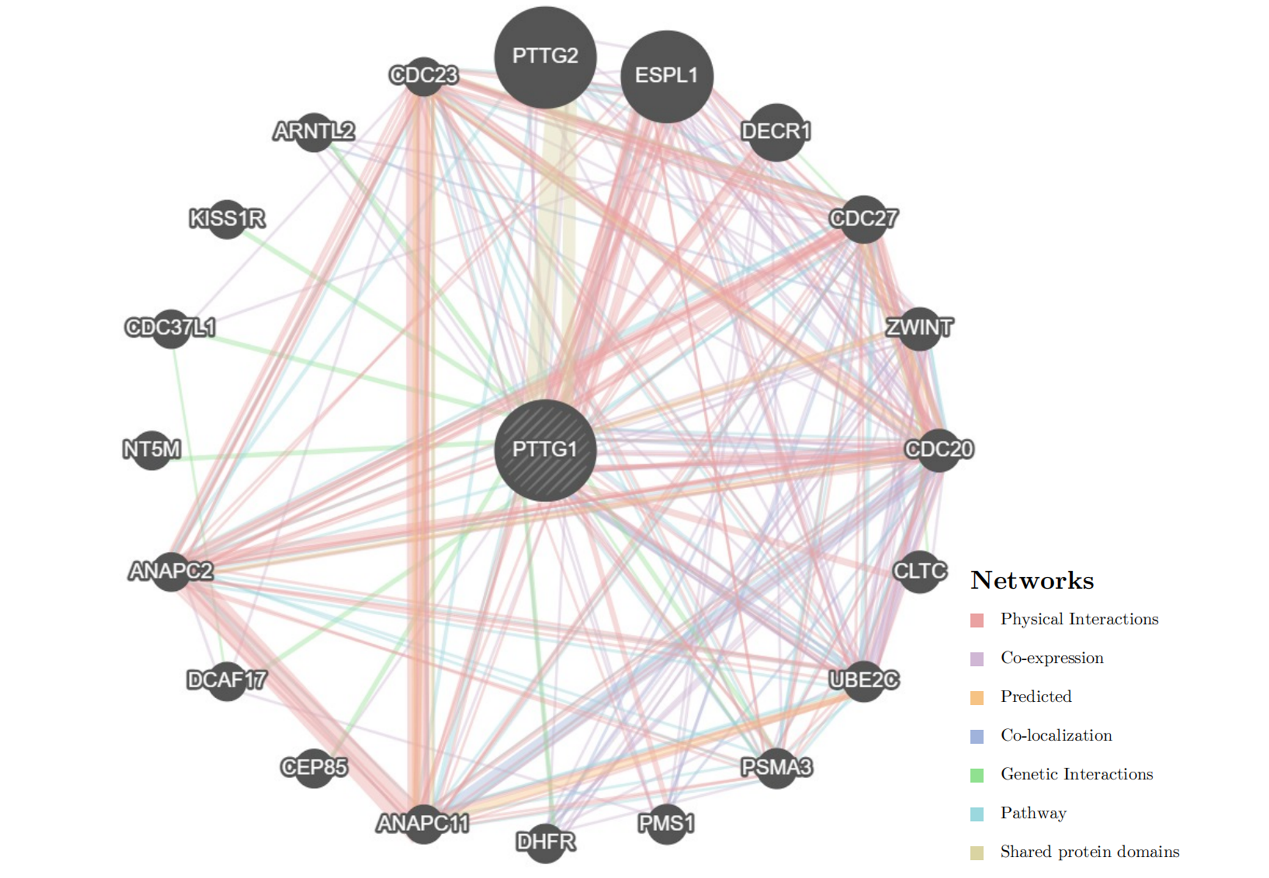


**Supplementary Figure S4.** Network of PTTG1 was analyzed by GeneMANIA.

## Supplementary Tables

**Supplementary table S1.** The tumor types and corresponding abbreviations.

| Abbreviations | Tumor |
| --- | --- |
| ACC | Adrenocortical carcinoma |
| BLCA | Bladder Urothelial Carcinoma |
| BRCA | Breast invasive carcinoma |
| CESC | Cervical squamous cell carcinoma and endocervical adenocarcinoma |
| CHOL | Cholangiocarcinoma |
| COAD | Colon adenocarcinoma |
| DLBC | Lymphoid Neoplasm Diffuse Large B-cell Lymphoma |
| ESCA | Esophageal carcinoma |
| GBM | Glioblastoma multiforme |
| GBMLGG | Glioma (GBM+LGG) |
| HNSC | Head and Neck squamous cell carcinoma |
| KICH | Kidney Chromophobe |
| KIPAN | Pan-kidney cohort (KICH+KIRC+KIRP) |
| KIRC | Kidney renal clear cell carcinoma |
| KIRP | Kidney renal papillary cell carcinoma |
| LAML | Acute Myeloid Leukemia |
| LGG | Brain Lower Grade Glioma |
| LIHC | Liver hepatocellular carcinoma |
| LUAD | Lung adenocarcinoma |
| LUSC | Lung squamous cell carcinoma |
| MESO | Mesothelioma |
| OV | Ovarian serous cystadenocarcinoma |
| PAAD | Pancreatic adenocarcinoma |
| PCPG | Pheochromocytoma and Paraganglioma |
| PRAD | Prostate adenocarcinoma |
| READ | Rectum adenocarcinoma |
| SARC | Sarcoma |
| SKCM | Skin Cutaneous Melanoma |
| STAD | Stomach adenocarcinoma |
| TGCT | Testicular Germ Cell Tumors |
| THCA | Thyroid carcinoma |
| THYM | Thymoma |
| UCEC | Uterine Corpus Endometrial Carcinoma |
| UCS | Uterine Carcinosarcoma |
| UVM | Uveal Melanoma |
